# Whole genome sequence data of a cellulose degrading bacterium, Bacillus sp. ABR isolated from the soils of Kurseong, India

**DOI:** 10.1016/j.dib.2026.113090

**Published:** 2026-07-21

**Authors:** Binoy Kumar Show, Tushar Lodha, Anudeb Ghosh, Prashant Dhakephalkar, Andrew B. Ross, Shibani Chaudhury, Srinivasan Balachandran

**Affiliations:** a Department of Environmental Studies, Siksha-Bhavana, Visva-Bharati, Santiniketan 731235, West Bengal, India; b Department of Chemical Engineering, Indian Institute of Technology Guwahati, Guwahati, Assam 781039, India; c Bioenergy Group, Agharkar Research Institute, Gopal Ganesh Agarkar Road, Pune 411004, Maharashtra, India; d School of Chemical and Process Engineering, University of Leeds, Leeds, LS2 9JT, United Kingdom

**Keywords:** Cellulase, Enzyme, Lignocellulose, Bacillus sp. ABR

## Abstract

The following study details the draft genome sequencing of a *Bacillus* sp. ABR isolated from Kurseong soil. *Bacillus* sp. ABR synthesizes cellulase, essential for the breakdown of plant biomass. It is a prospective supply of enzymes significant for digestion, particularly lignocellulases. Genomic DNA was extracted from a pure bacterial colony (expanded in liquid culture before extraction) with a QIAGEN Blood and Tissue Kit (QIAgen Inc., Canada). DNA sequencing was carried out on the Illumina HiSeq X platform employing 2 × 150 bp paired-end chemistry, yielding approximately 1.90 Gb of high-quality sequence data. The raw data were assembled into 12 contigs exceeding 1000 base pairs, with a sequence coverage of 509X (N50 value = 901,304 and L50 = 2). The assembled genome consists of 3,729,524 base pairs and exhibits a mean GC content of 41.43%. Functional annotation using the RAST server predicted 3,927 coding sequences (CDSs), 59 RNA coding genes, and 3 rRNA genes. The strain ABR harbours a diverse repertoire of Carbohydrate-Active Enzymes (CAZymes), confirming the strain's cellulolytic potential, which has application in lignocellulosic biomass degradation.

Specifications Table

**Table:** 

Subject	Microbiology • Applied Microbiology
Specific subject area	Omics: Genomics
Type of data	Table, Figure Raw, Analyzed, Filtered, Deposited
Data collection	Genomic DNA was extracted from a single bacterial colony (expanded in liquid culture before extraction) using the QIAgen Blood and Tissue Kit (QIAgen Inc., Canada). Library preparation and sequencing were conducted on the Illumina HiSeq X platform. Following sequencing, Cutadapt v2.9 was used for size selection, trimming, and quality control using FastQC v0.11.9. The genome was de novo assembled using Unicycler v0.4.8, with assembly summary statistics performed by QUAST v5.2.0. The genome assembly was carried out using Unicycler v0.4.8. The genome annotation was performed by the Rapid Annotation using Subsystem Technology (RAST) server.
Data source location	The ABR strain was isolated from the soil samples collected near Kurseong in West Bengal, India (26.52’49” N latitude and 88.16’13” E longitude).
Data accessibility	Repository name: NCBI (National Center for Biotechnology Information) Data identification number: BioProject accession number PRJNA890881; The Biosample number is SAMN31300158; The Sequence Read Archive (SRA) accession number is SRR21915758 Direct URL to data: https://www.ncbi.nlm.nih.gov/bioproject/PRJNA890881 https://www.ncbi.nlm.nih.gov/sra/SRR21915758

## 1 Value of the Data

• The draft genome sequence of *Bacillus* sp. strain ABR provides a useful resource for exploring bacterial ecology and taxonomy, particularly in areas related to species identification and distribution.
• The genomic study reveals the genetic capabilities of *Bacillus* sp. ABR to produce a variety of CAZymes and showcase its potential in lignocellulosic biomass valorization.
• The dataset presented in this study will interest researchers in fields such as environmental microbiology, environmental biotechnology, genomics, and renewable energy.
• The genomic information of *Bacillus* sp. strain ABR can support comparative genomic studies across different bacterial strains and environmental niches.

## 2 Background

*Bacillus sp.* ABR is a member of the *Bacillaceae* family. Gram-positive, rod-shaped microorganisms of this genus are extensively distributed in soil and are renowned for their ability to withstand harsh environmental conditions [1]. The lignocellulosic nature, structural rigidity, and hydrophobic properties of organic waste and invasive vegetation biomass, which are characterized by numerous stable chemical linkages, frequently impede the efficient anaerobic digestion (AD) of these materials [2]. Lignocellulosic biomass consists of polymeric components, including cellulose, hemicellulose, and lignin. Cellulose constitutes the primary fraction of lignocellulose, embedded within a hemicellulose matrix and encased by an outer lignin barrier [3]. The presence of a variety of microorganisms that produce potent enzymes capable of degrading lignocellulose has been reported in numerous studies on microbial degradation [4]. Investigating these microbial consortia is ecologically significant due to the direct enzymatic degradation of lignocellulosic compounds by soil microorganisms across a wide range of pH and temperature conditions. The present investigation concentrates on the genomic analysis and sequencing of *Bacillus sp.* strain ABR, which is capable of producing cellulase.

## 3 Data Description

**Fig. 1:**
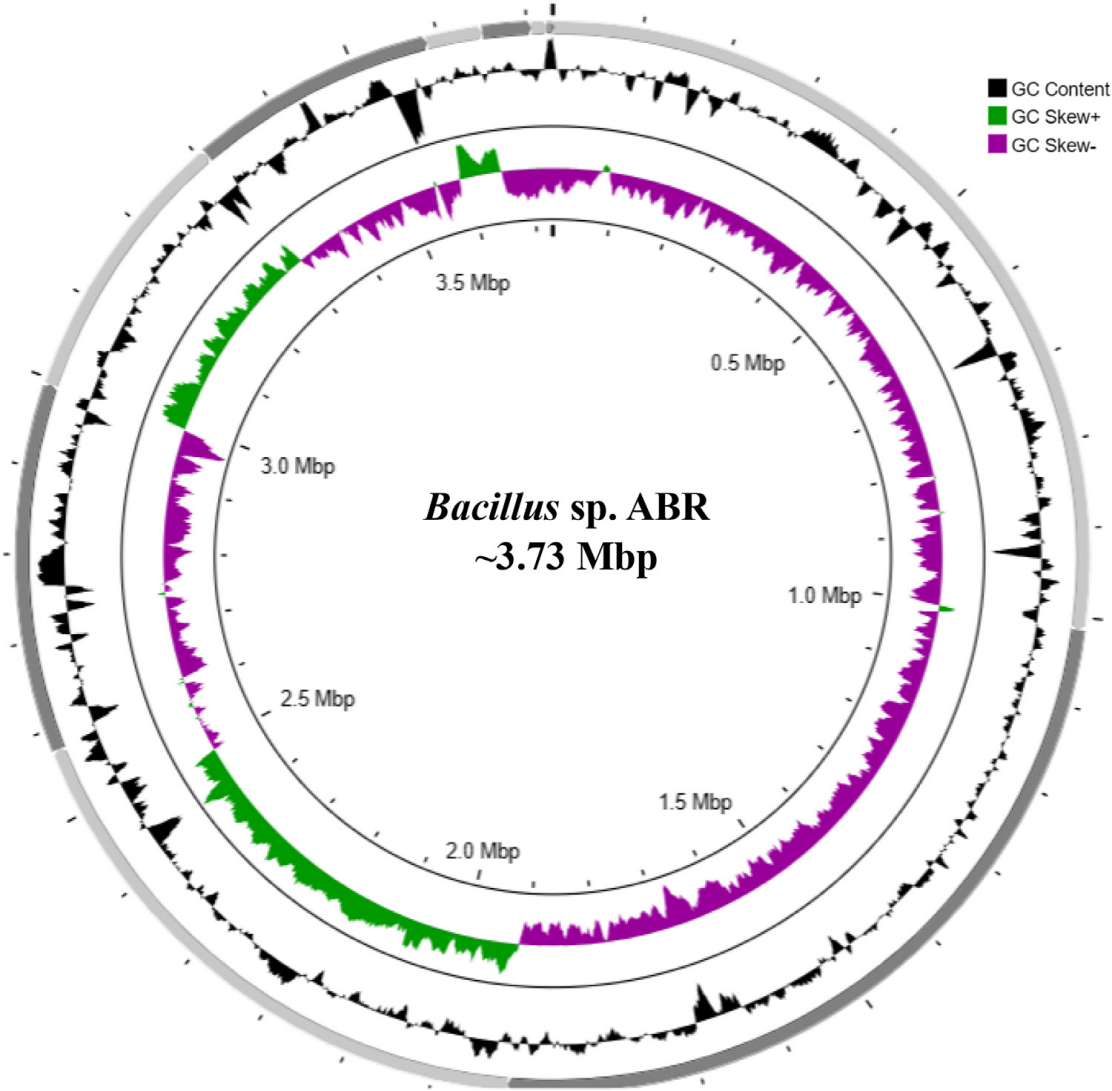
Circular representation of *Bacillus* sp. ABR genome (∼3.73 Mbp). The genome map is generated using the CG view server.

This article presents the complete genome sequence of *Bacillus* sp. strain ABR, which harbors lignocellulase-related genes responsible for plant biomass degradation. The assembled genome consists of 12 contigs, exhibiting an N50 value of 901,304 bp and an L50 of 2. The assembly demonstrates high quality, showing 100% completeness, zero contamination, and a sequencing depth of 509X. The total genome length is 3,729,524 bp, with an average GC content of 41.43% (Fig. 1). Genome annotation identified 3,927 coding sequences (CDS), 58 tRNA genes, and 3 rRNA genes (Table 1).

**Table 1:** General genomic features of *Bacillus* sp. *ABR*

Feature	Value
Genome size (bp)	3,729,524
G+C content (%)	41.43
No. of contigs	12
N50	901,304
N90	278,819
L50	2
L90	6
CDS (coding sequences)	3,927
tRNA	58
rRNA	3

**Fig. 2:**
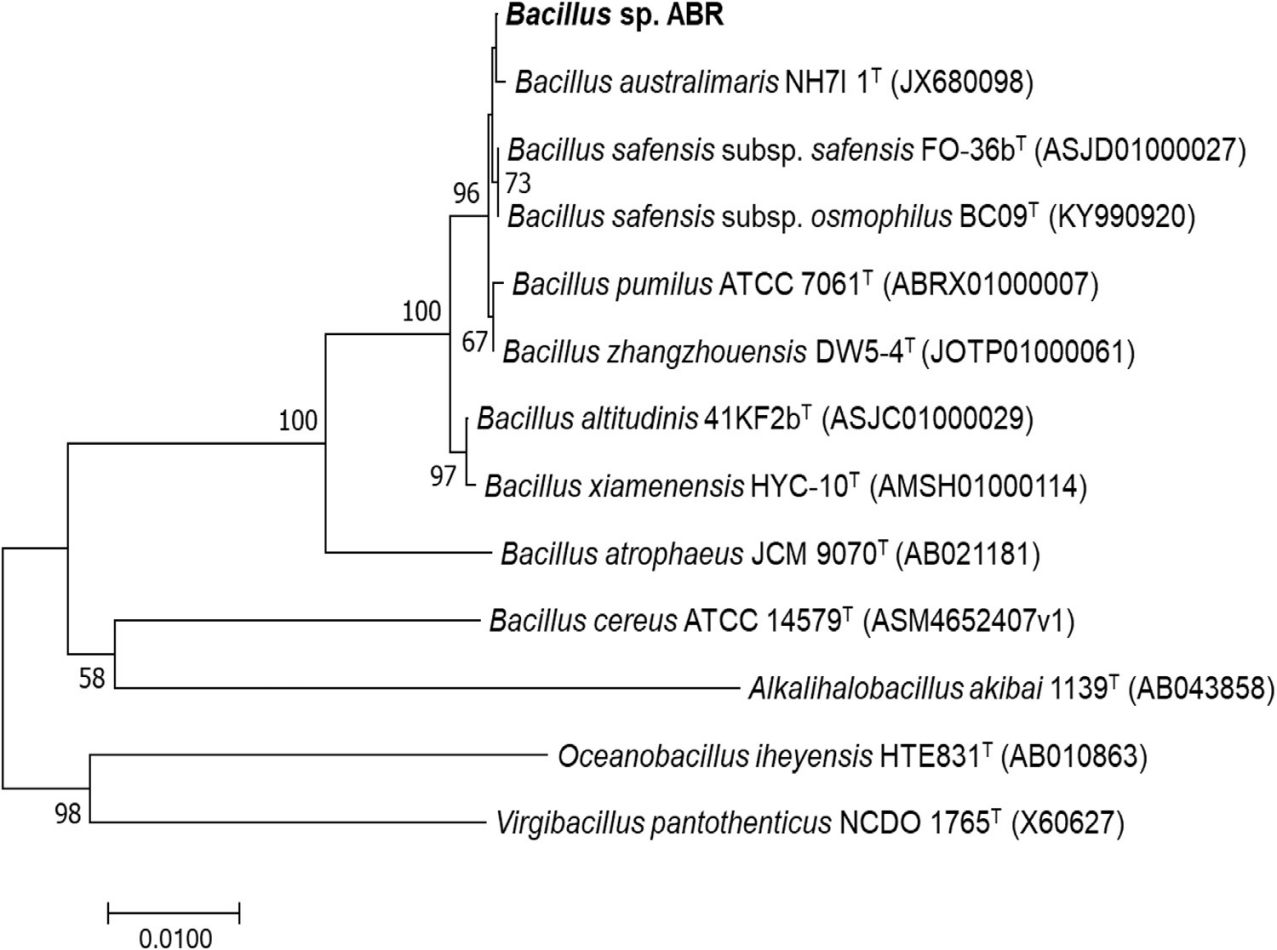
Phylogenetic tree based on 16S rRNA gene sequence showing the phylogenetic relationship between the members of the genus *Bacillus*. The tree was constructed using MEGA X and *Alkalihalobacillus akibai* 1139T (AB043858), *Oceanobacillus iheyensis* HTE831^T^ (AB010863) and *Virgibacillus pantothenticus* NCDO 1765^T^ (X60627) was used as an outgroup. The 16S rRNA gene bank accession number is shown in parentheses. The bootstrap percentage refers to Neighbor joining analysis. The bootstrap values above 50 for each node are indicated. Scale bar indicates the number of substitutions per site.

**Fig. 3:**
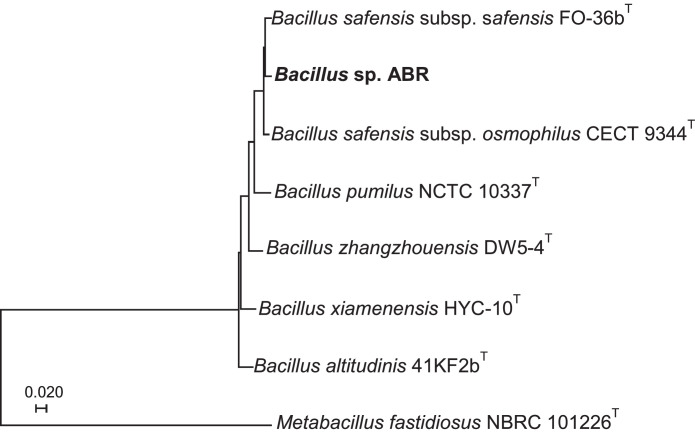
Phylogenetic tree constructed using the genome of *Bacillus* sp. ABR and related members with the 92 bacterial core gene sequences. The 92 gene sequences were extracted using the Up-to-date bacterial core gene tool (UBCG), which is a widely used resource for delineating the phylogeny of bacteria, and the phylogenetic tree was constructed using MEGA X with NJ and ME algorithms. Bar, 0.02 nucleotide substitution per position.

The 16S rRNA gene sequence of strain ABR exhibited over 99% identity with multiple members of the *Bacillaceae* family, encompassing seven Bacillus species. The nearest phylogenetic cousin was determined to be *Bacillus safensis subsp. safensis*, exhibiting 99.93% sequence similarity. In the maximum-likelihood phylogenetic tree derived from the 16S rRNA gene, strain ABR grouped into the same lineage as *Bacillus safensis subsp. safensis* (Fig. 2). Moreover, whole-genome phylogenetic analysis revealed a strong relationship between *Bacillus sp.* ABR and *Bacillus safensis subsp. safensis* (Fig. 3). The average percentage of nucleotide identity (ANI) between strain ABR and *Bacillus safensis subsp. safensis* strain FO-36b was 96.27%, while its digital DNA–DNA hybridization (dDDH) value was 68.30%, thereby validating its classification as *Bacillus sp.* strain ABR.

**Fig. 4:**
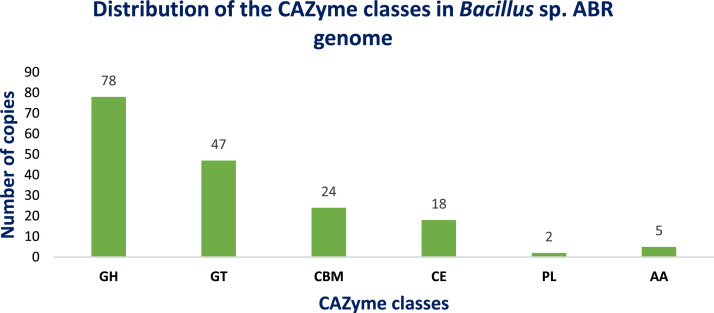
The distribution of the major CAZyme families in the genome of *Bacillus* sp. ABR. GH-Glycoside Hydrolases; GT-Glycosyl Transferases; CBM-Carbohydrate-Binding Modules; CE-Carbohydrate Esterases; PL-Polysaccharide Lyases; and AA-Auxiliary Activities. The prevalence of families such as GH1, GH3, GH5, and GH9 correlates with the isolate's observed cellulolytic activity.

The strain ABR exhibits the cellulose-degrading capabilities. The genome was screened for the presence of CAZymes gene using the dbCAN server. The CAZyme analysis shows that the *Bacillus* sp. ABR exhibits a robust genetic potential for polysaccharide degradation. The genome shows the presence of various glycoside hydrolases (GHs) such as GH1, GH2, GH3, GH4, GH5, GH9, GH13, GH16, GH23, GH28, GH43, GH48, GH51, GH53, GH73 and other GH families. These glycoside hydrolases are responsible for the celluloase and hemicellulose degradation (Fig. 4). The CAZyme analysis also reveals the presence of the cellulose-degrading families like GH9 and GH48, along with the carbohydrate-binding modules (CBMs) in strain ABR. The presence of the signal peptide in several enzymes implies that the strain has a genetic capability for an extracellular secretion system for polysaccharide hydrolysis. The genome also reveals the presence of Polysaccharide Utilizing Loci (PULs) targeting cellulose beta-glucan and chitin, indicating the capacility of complex carbohydrate metabolism of strain ABR. The CAZyme arsenal corroborates the physiological trait of cellulolytic capability of *Bacillus* sp. ABR and its ecological role in soil organic matter turnover in the Kurseong region.

## 4 Experimental Design, Materials, and Methods

### 4.1 Isolation of *Bacillus* sp. strain ABR

The ABR strain was isolated from the soil samples collected near Kurseong in West Bengal, India (26.52’49” N latitude and 88.16’13” E longitude). A total of five soil samples were collected from sites exhibiting organic matter accumulation and visible litter decomposition, suggesting potential cellulolytic microbial habitats. Samples were taken from a depth of 5–10 cm using sterile tools, placed in sterile polyethylene bags, and transported to the laboratory for bacterial isolation. Isolation was performed using the conventional serial dilution technique, and colonies were grown on Bushnell-Haas agar supplemented with 1% (w/v) carboxymethylcellulose (BH-CMC) as the sole source of carbon used to separate cellulolytic bacteria. The isolate was subsequently cultivated in Bushnell Haas agar containing 1% CMC and incubated for 72 hrs at 37°C. To ensure purity, repeated streaking on BH-CMC agar plates was carried out and maintained at 4°C [5].

### 4.2 Genomic DNA extraction

Genomic DNA was extracted from the bacterial culture following the manufacturer’s instructions using the Qiagen Blood and Tissue Kit (Qiagen Inc., Canada). The purity, assessed by the OD_260/280_ ratio, and the DNA concentration were determined with an Agilent BioTek Epoch 2 microplate reader (USA) [6].

### 4.3 Whole genome sequencing and assembly

Whole-genome sequencing of strain ABR was carried out on the Illumina HiSeq X platform employing 2 × 150 bp paired-end chemistry, yielding approximately 1.90 Gb of high-quality sequence data. Preliminary quality assessments of the sequences were conducted using FastQC v0.11.9 (https://www.bioinformatics.babraham.ac.uk/projects/fastqc/) [7]. Genome assembly was performed utilizing Unicycler v0.4.8 with default settings, integrating BayesHammer for error correction [8]. The quality of the draft genome was assessed utilizing CheckM v1.1.6 and QUAST v5.1.0 [9,10]. A circular representation of the draft genome was generated using the CGView server (https://www.cgview.ca) [11] (Fig. 1). Annotation and predictions of genes were conducted using the RAST server. The CAZyme analysis was conducted on the dbCAN3 server [12].

### 4.4 Phylogenetic analysis

The EzBioCloud service (www.ezbiocloud.net) was used to retrieve the virtually complete 16S rRNA gene sequence from the whole genome and compare it with type strains [13]. The NCBI database (www.ncbi.nlm.nih.gov) provided sequences from closely related type taxa, which were then aligned using MEGA X's MUSCLE method [14]. The Tamura 3-parameter model with Gamma distribution and invariant sites (T92+G+I) was used for nucleotide substitution modeling, and the complete deletion option was used to fill in gaps and missing data [15]. 1000 bootstrap replications were used in MEGA X to create maximum-likelihood phylogenetic trees (Fig. 2). Genome-to-genome distances (GGDs) and digital DNA-DNA hybridization (dDDH) were included in the whole genome-based taxonomy analysis [16]. The phylogenetic tree based on the genome was constructed utilizing the UBCG tool (Fig. 3) [17].

## Limitations

Not applicable.

## Ethics Statement

The authors have read and follow the ethical requirements for publication in Data in Brief and confirming that the current work does not involve human subjects, animal experiments, or any data collected from social media platforms.

## CRediT Author Statement

**Binoy Kumar Show**: Conceptualization, Data curation, Formal analysis, Methodology, Validation, Writing – original draft. **Tushar Lodha**: Conceptualization, Data curation, Formal analysis, Methodology, Resources, Writing – original draft. **Anudeb Ghosh**: Data curation, Formal analysis. **Prashant Dhakephalkar**: Data curation, review. **Andrew B. Ross**: Conceptualization, Investigation, Project administration, Supervision, Writing – review and editing. **Shibani Chaudhury**: Conceptualization, Investigation, Project administration, Supervision, Writing – review and editing. **Srinivasan Balachandran**: Conceptualization, Investigation, Project administration, Supervision, Writing – review and editing.

## Data Availability

National Center for Biotechnology InformationWhole genome sequence data of a cellulose degrading bacterium, Bacillus sp. ABR isolated from the soils of Kurseong, India (Original data)
